# Synthesis and Characterization of PLA-Micro-structured Hydroxyapatite Composite Films

**DOI:** 10.3390/ma13020274

**Published:** 2020-01-08

**Authors:** Andreea Madalina Pandele, Andreea Constantinescu, Ionut Cristian Radu, Florin Miculescu, Stefan Ioan Voicu, Lucian Toma Ciocan

**Affiliations:** 1 Advanced Polymer Materials Group, Faculty of Applied Chemistry and Material Science, University Polytehnica of Bucharest, str. Gheorghe Polizu 1-7, 011061 Bucharest, Romania; pandele.m.a@gmail.com (A.M.P.); radu.ionut57@yahoo.com (I.C.R.); 2 Faculty of Applied Chemistry and Materials Science, University Politehnica of Bucharest, Gheorghe Polizu 1-7, 011061 Bucharest, Romania; 3 Faculty of Materials Science, University Politehnica of Bucharest, Splaiul Independentei 313, 011061 Bucharest, Romania; andreeaelena01c@gmail.com (A.C.); f_miculescu@yahoo.com (F.M.); 4 “Carol Davila” University of Medicine and Pharmacy, Prosthetics Technology and Dental Materials Department, 37, Dionisie Lupu Street, District 1, 020022 Bucharest, Romania; tciocan@yahoo.com

**Keywords:** polylactic acid, hydroxyapatite, composite films

## Abstract

This article presents a facile synthesis method used to obtain new composite films based on polylactic acid and micro-structured hydroxyapatite particles. The composite films were synthesized starting from a polymeric solution in chloroform (12 wt.%) in which various concentrations of hydroxyapatite (1, 2, and 4 wt.% related to polymer) were homogenously dispersed using ultrasonication followed by solvent evaporation. The synthesized composite films were morphologically (through SEM and atomic force microscopy (AFM)) and structurally (through FT-IR and Raman spectroscopy) characterized. The thermal behavior of the composite films was also determined. The SEM and AFM analyses showed the presence of micro-structured hydroxyapatite particles in the film’s structure, as well as changes in the surface morphology. There was a significant decrease in the crystallinity of the composite films compared to the pure polymer, this being explained by a decrease in the arrangement of the polymer chains and a concurrent increase in the degree of their clutter. The presence of hydroxyapatite crystals did not have a significant influence on the degradation temperature of the composite film.

## 1. Introduction

Polymeric films represent a particular domain of materials due to their controlled and directed selectivity capacity [1,2]. Recently, besides the common applications which imply the separation of constituents from a complex composition [3,4], less conventional applications such as adjuvant films for osteointegration have also been studied. In this case, the membrane can be placed at the interface between a metal implant and bone. These materials aim to improve and to accelerate the integration of the metal implant into the bone [5,6,7]. Composite membranes and polymeric films based on hydroxyapatite (HA) have lately seen great development, especially due to their potential applications in orthopedics [8]. Among the most commonly used polymers employed to obtain the aforementioned composites, biocompatible and bioresorbable polymers are usually preferred due to their ability to be desorbed over time into the human body and to promote osteoblast proliferation during bone implant welding. Hence, composites films based on hydroxyapatite and various polymers such as cellulose derivatives [9], starch [10], and polylactic acid [11] have been synthesized. Polylactic acid (PLA) is a synthetic polymer that can have both a crystalline [12] and an amorphous [13] structure. There are several studies which have indicated the use of PLA/HA composites for different applications [14]. Composites of PLA/HA with a percentage of 80 wt.% HA loading have been synthesized by adding filler into the polymer melt [15]. The obtained composites have been investigated in terms of their mechanical properties, and a significant improvement of Young’s modulus has been observed. Moreover, the reported values are close to those of natural bone. In order to synthesize composite films with improved mechanical and thermal properties, new composite films based on polylactic acid/hydroxyapatite/graphene oxide (PLA/HA/GO) have also been prepared. In this case the HA and GO have been homogenously dispersed into a polymer solution using dimethylformamide and methylene chloride as solvents. The final composite films were obtained after the evaporation of the solvents at 40 °C [16]. The as-synthesized composite films were tested for potential use in tissue regeneration [17]. It seems that the presence of GO promotes the dispersion of HA into the polymer matrix and also has a positive impact on the biocompatibility of the final material.

Due to PLA’s good biocompatibility [18] and bioresorbability [19], polymer composites with HA nanoparticles are focused on the synthesis of materials that improve the osteointegration of the implantable scaffold. Rakmae et al. have reported the modification of the surface of HA particles with 3-aminopropyltriethoxysilane (APES) or 3-methacryloxypropyltrimethoxysilane (MPTS) to increase the compatibility between the inorganic filler and the polymer matrix [20]. The researchers observed that the surface modification of HA nanoparticles significantly improved the mechanical and thermal properties of the synthesized composites, preventing the cleavage of PLA chains and increasing the degree of biocompatibility of the final material. At the same time, the mechanical and thermal resistances were further improved by adding a third polarizer to the synthesized composites, namely, poly-caprolactone [21]. The presence of poly-caprolactone allows an easier adjustment of some properties of the membrane by controlling the pore diameter and distribution.

Furthermore, PLA polymeric composites with very high mechanical strength have been synthesized by adding epoxy resin to the composite matrix [22]. Superior mechanical properties were observed in the case of the addition of HA and silver nanoparticles within a melted PLA matrix [23]. The use of this synthesis procedure allows for the obtaining of composites with a high percentage of hydroxyapatite (18 wt.%), and, at the same time, the existence of silver particles provides excellent antibacterial properties. High antibacterial activity for *Escherichia coli* and moderate antibacterial activity for *Staphylococcus aureus* have been observed. Generally, all synthesized composites have exhibited a non-cytotoxic character and can be used both in vitro and in vivo [24,25,26]. The optimal mechanical properties for implantable materials based on PLA/HA have been achieved for composite materials with a very high content of HA (70–80 wt.%) [15]. These percentages are conditioned by a few synthetic methods that can lead to uniform materials which imply such high inorganic filler content.

The current article presents an easy method with which to synthesize compact PLA/HA composite films by dispersing various concentrations of micro-structured HA particles in PLA solution using chloroform as a solvent, followed by evaporation. The obtained composite films were characterized by SEM microscopy, atomic force microscopy (AFM) microscopy, FT-IR spectroscopy, Raman spectroscopy, and thermal analysis. The novelty degree of the present research is given by the synthesis method used, which involves solvent evaporation for obtaining the films, the use of chloroform as a solvent (with a low boiling point and rapid formation of films by evaporation of the solvent) and also the use of the biogenic source HA.

## 2. Materials and Methods

### 2.1. Synthesis of Hydroxyapatite Particles

The hydroxyapatite particles were synthesized from spongy bone samples which were obtained after removal of the cortical components in accordance with a previously described procedure reported in the literature [27,28,29]. Firstly, bovine bone samples were mechanically cleaned, followed by a heat treatment at 500 °C for one hour in order to remove organic components. Proper thermal treatment was performed at 1200 °C for 6 h with a heating rate of 10 °C/min. After cooling in the air, the samples were ground in an agate ball mill and then sieved. For the present study, hydroxyapatite particles with sizes smaller than 40 µm were used.

### 2.2. Synthesis of PLA/HA Composite Films

Polylactic acid was dissolved in chloroform at a concentration of 12 wt.%. The hydroxyapatite particles were dispersed by ultrasonication for 30 min into the polymer solution at three different concentrations (1, 2, and 4 wt.%, respectively) in order to obtain a homogenous solution. The composite films were prepared after casting the obtained mixture into a Petri glass 60 mm in diameter (LabBox, Barcelona, Spain) and evaporating the solvent at 40 °C for 72 h in an oven. The long evaporation time was necessary due to the low porosity of the obtained films and the slower solvent evaporation during polymer precipitation. After synthesis, the composite films were washed with distilled water and ethanol to remove any chloroform and polymer residues and kept dry until use.

### 2.3. Characterization of Obtained Materials

FT-IR spectra were recorded on a Bruker VERTEX 70 spectrometer (Bruker, Massachusetts, United States) using 32 scans with a resolution of 4 cm^−1^ in the 4000–600 cm^−1^ region. The samples were analyzed using Attenuated Total Reflectance annex (ATR) (Bruker, Massachusetts, United States).

Raman spectra were registered on a DXR Raman Microscope (Thermo Fischer, Waltham, Massachusetts, USA) from Thermo Scientific using a 532 nm laser line and a number of 10 scans. The laser beam was focused using the 10× objective of the Raman microscope.

Scanning electron microscopy was performed on a FEI XL 30 ESEM TMP microscope equipped with an EDAX Sapphire device (FEI/Philips, Hillsboro, OR, USA).

Thermogravimetric analysis (TGA) curves were registered on Q500 TA Instruments (TA Instruments, New Castle, DA, USA) equipment using a nitrogen atmosphere from room temperature to 800 °C and a heating rate of 10 °C/min.

Differential scanning calorimetry (DSC) curves were recorded using Netzsch DSC 204 F1 Phoenix equipment (Netzsch, Selb, Germany). The sample was heated from room temperature (RT) to 300 °C using a heating rate of 5 °C/min under nitrogen (20 mL/min flow rate).

AFM analyses were performed using a multimode apparatus Agilent 5500 (Santa Clara, California, United States) equipped with an AC mode III controller. The contact mode images were obtained using a triangular silicon nitride cantilever with a typical spring constant of 0.06 N/m and a resonant frequency of 10 kHz. This type of cantilever is a very soft one and is specific for contact mode and used to obtain images without damaging the sample. The analyses were performed under ambient conditions with a calibrated piezo-scanner with a maximum xy range of 90 µm × 90 µm and a z scan range of 7 µm.

Mechanical tests were performed according to the European Standard EN ISO 527-3 part 3 (for films and tests) using a universal mechanical tester (Instron, Model 3382, Massachusetts, USA) at a relative humidity of ~50 % and a speed of 3 mm/min. The dimensions of the samples were 10 cm × 1 cm × 0.038 cm. For each composite film a minimum of five specimens were tested and the average values and standard deviation (±SD) were reported. 

## 3. Results and Discussion

**Figure 1:**
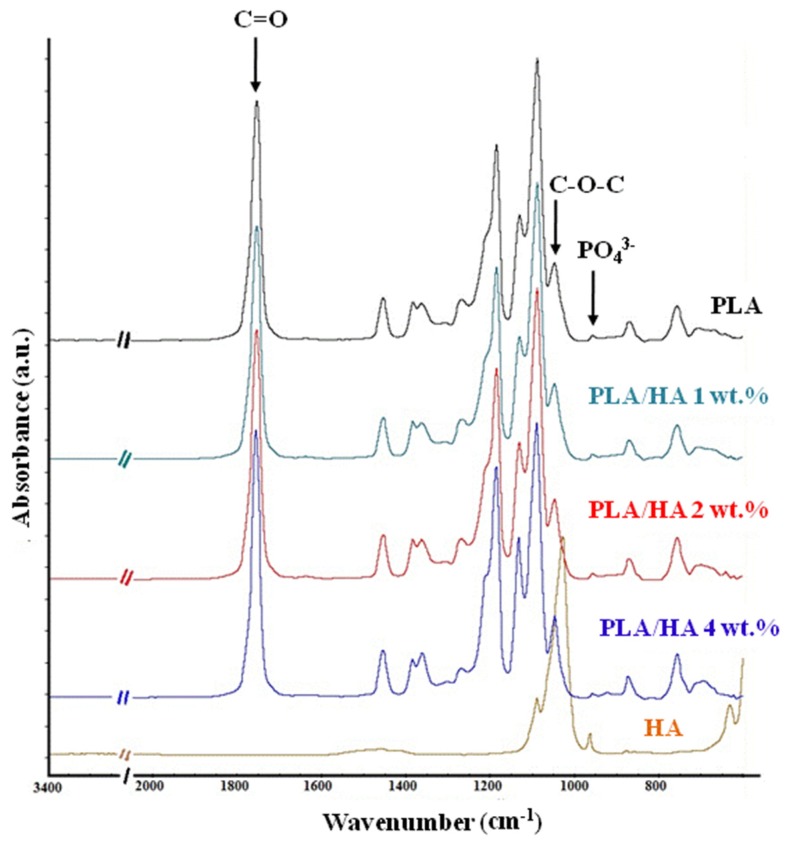
FT-IR spectra of hydroxyapatite (HA) and polylactic acid (PLA)/HA composite films.

Figure 1 presents the FT-IR spectra of the HA, PLA, and PLA/HA composite films. In the HA spectrum, the bands corresponding to the stretching vibration of PO_4_^3−^ are observed at 603 cm^−1^, 957 cm^−1^, and 1041 cm^−1^ respectively. In the PLA spectrum, the bands at 2998 cm^−1^ and 2947 cm^−1^ can be attributed to the asymmetrical and symmetrical stretching vibrations of the C–H bond, the band at 1755 cm^−1^ can be attributed to the stretching vibrations of the C=O bond, the band at 1457 cm^−1^ can be attributed to the deformation vibration of the CH_3_ group, the bands at 1184 cm^−1^ and 1089 cm^−1^, respectively, can be assigned to the C–O–C binding vibrations, and the band at 871 cm^−1^ corresponds to the vibrations of the C–COO bond [30]. The spectra of the composite membranes are similar to the PLA spectrum, exhibiting only the vibration bands corresponding to the polymer structure. This is due, on the one hand, to the overlapping of the vibration bands of HA in the range of 600–1100 cm^−1^ with the bands corresponding to the polymer, and on the other hand because of the small amount of phosphate groups present in the composite film composition compared to the C–H bonds from the polymer structure [9]. However, slight changes in the peak intensity can be identified. Moreover, when considering as the reference band the band at 1088 cm^−1^ (ν_C–O–C_), whose intensity is unchanged across all the spectra, and calculating the ratio between this band and the band at 1045 cm^−1^ (δ_C–CH3_), it can be observed that there is an increase in the intensity ratio in the case of the composite films compared with the pure polymeric film. This could suggest the presence of some interferences between HA and the polymer chain [31].

**Figure 2:**
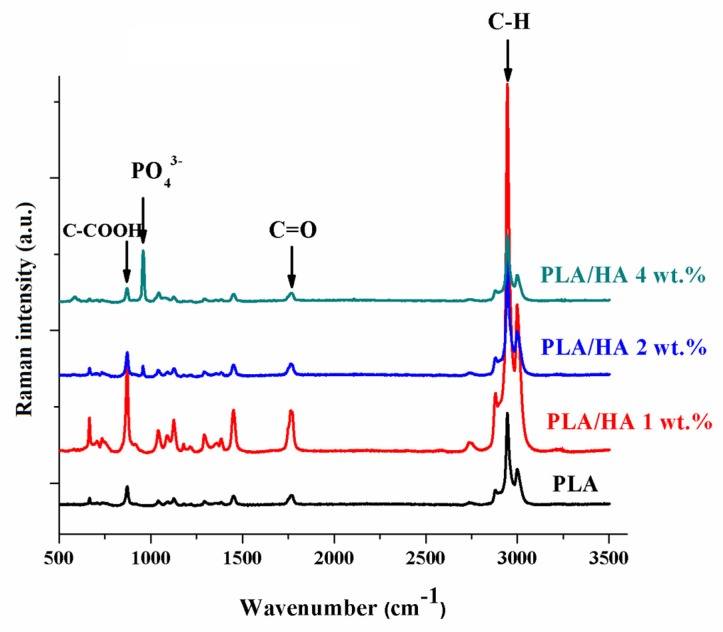
Raman spectra of PLA and PLA/HA composite films.

The presence of HA in the composite films was also observed using Raman spectroscopy. Figure 2 shows the Raman spectra for PLA and the PLA/HA composite films. According to the figure, the PLA spectrum presents characteristic bands observed at 2995 cm^−1^, 2990 cm^−1^, and 2878 cm^−1^, which can be attributed to asymmetric and symmetrical stretching vibrations of the C–H (ν_as/sCH3_) bond of the PLA chain; 1762 cm^−1^, which can be attributed to the stretching vibration of the C=O (ν_C=O_) bond; 1447 cm^−1^, which can be attributed to the asymmetric deformation vibration of the CH_3_ bound (δ_asCH3_); 887 cm^−1^, which can be attributed to the stretching vibration of the C–COO (ν_C–COO_) bond [16]. Compared to the PLA spectrum, the spectra of the composite films with 2 and 4 wt.% HA loaded onto them contain all the polymer-specific bands as well as the presence of an additional band around 960 cm^−1^ which corresponds to the PO_4_^3−^ stretching vibration. This band comes from the HA structure and represents a piece of quite solid evidence for the presence of the inorganic compound in the polymer matrix. The absence of this band in the case of the composite membrane’s spectrum with 1 wt.% HA loaded on is due to a small amount of HA being introduced into the polymer matrix which could be homogenously covered by the polymer. Moreover, for the membrane with 4 wt.% HA loaded onto it, due to the high amount of HA introduced into the polymer matrix, the presence of two additional bands at 585 cm^−1^ and 428 cm^−1^ which can be attributed to the asymmetrical deformation vibrations of PO_4_^3−^ in the HA structure can be observed [32].

**Figure 3:**
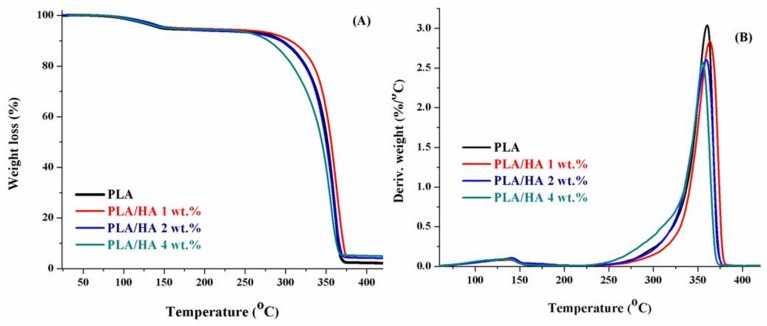
(**A**) Thermogravimetric analysis (TGA) and (**B**) DTG curves of PLA and PLA/HA composite films.

Thermogravimetric analysis was used to study the effect of HA nanoparticles on the thermostability of the polymer. Figure 3 illustrates the TGA and Differential Thermal Gravimetry (DTG) curves of PLA and the composite films with different concentrations of HA added. According to Figure 3, all membranes exhibit a similar profile with two degradation steps. The thermostability of the materials was tested by measuring the decomposition temperature at 10% mass lost (Td_10%_). An increase in the thermostability of the composite films with 1 and 2 wt.% HA loaded on was observed at about 53 °C compared to pure PLA. By further increasing the HA contents to 4 wt.%, the Td_10%_ was shifted to lower values; this was due to the formation of some HA agglomerates in the polymer matrix. However, the thermostability of the HA composite film with 4 wt.% HA can be seen to be superior to the pure polymer films, and this increase in thermal stability is due to the formation of strong hydrogen interactions and Van der Walls forces between inorganic particles and the polymeric chain during the homogenization process. More recent studies have suggested that when using higher concentrations of HA in the polymeric matrix it is very difficult to achieve a homogenous dispersion of inorganic filler into the matrix, leading to the formation of aggregates and diminishing the shielding effect of the particles [33]. On the other hand, the maximum degradation temperature of the first degradation step is almost invariable and the decomposition temperatures report the same value for all the samples (approximately 350 °C).

**Figure 4:**
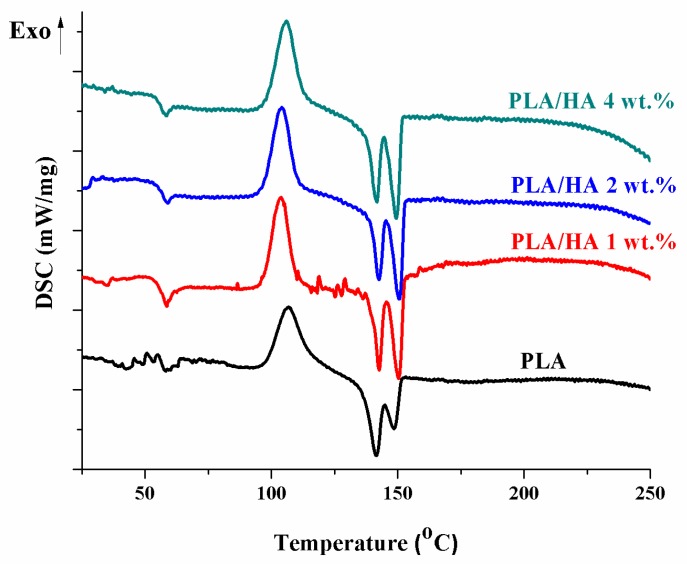
Differential scanning calorimetry (DSC) curves of PLA and PLA/HA composite films.

The thermal properties of the materials were also studied by DSC. Figure 4 shows the DSC curves for PLA and the composite films with 1, 2, and 4 wt.% HA added. The results obtained from the DSC curves for both the pure polymer and the composite films are summarized in Table 1. According to the figure, all samples presented two endothermic peaks around 142 °C and 150 °C due to the gradual melting of different sized polymer blades [31]. Moreover, on the DSC curves the presence of an exothermic peak around 106 °C can be observed which corresponds to the crystallinity of the polymer (a cold crystallization—temperature of crystallization—Tc). After intercalation of HA particles to the polymeric matrix, the Tc values tends to decrease due to the fact that HA particles act as nucleation centers for PLA crystals. Similar results have been reported in the literature by Maria Persson et al. [34].

The degree of crystallinity was further calculated according to the equation below, assuming an ideal melt heat of 93.7 J (Equation (1)).

(1) $$\chi_{c}=100\times \left(\Delta H_{m}-\Delta H_{c}\right)/93.7$$

After the calculations, values of crystallinity percentage ranging between 3 and 0.7% were obtained. It can be observed that at a low HA concentration (1 wt.%) the crystallinity of the composite films decreased significantly compared to the pure polymer. By increasing the amount of HA added, the value began to increase, reaching 1.7% for the composite by 4 wt.%. However, this value was below the crystallinity obtained in the case of the pure polymer. This could be explained by the fact that the presence of a small amount of HA (less than 4 wt.%) leads to a decrease in the crystallinity of the polymer as the presence of HA decreases the orientation of the polymer chains by increasing their degree of disorder. Furthermore, it appears that the presence of HA had no significant effect on the melting temperature, but a slight decrease indicates that the crystal size was less stable.

From the DSC assays we can conclude that the addition of HA particles in the polymeric matrix has an influence on the polymer chain arrangement, which further leads to a change in the polymer behavior when heated. This is due to the fact that HA particles act as nucleation centers in order to obtain a rigid phase that has a significant effect on the final polymer properties.

**Figure 5:**
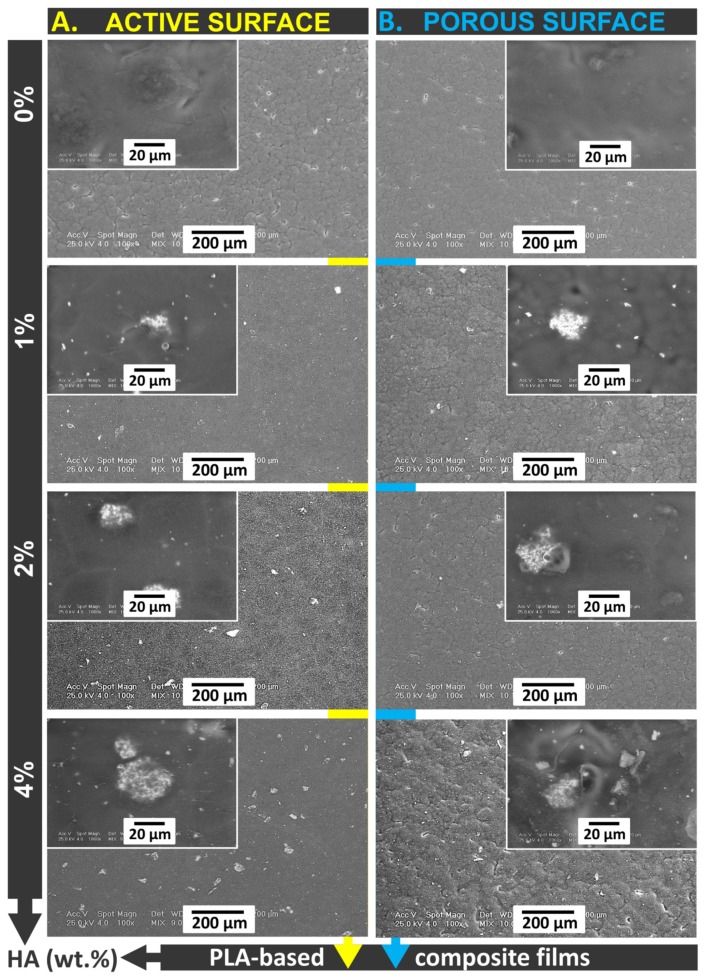
SEM images of PLA and PLA/HA composite films. (**A**) Active surface of the membranes, (**B**) porous surface of the films.

The presence of HA in the polymer matrix has also been shown to have an effect on the morphology of composite films. Scanning electron microscopy (Figure 5) revealed some differences between the analyzed samples. The pure PLA film displayed a smooth surface with no polymeric formation.

The pores were shown to have very small diameters but a difference was observed between one side and the other, indicating the asymmetry of the membrane. Contrary to polymeric membranes obtained by phase inversion (precipitation with a non-solvent), the films obtained by solvent evaporation can be seen to have much smaller pores due to the slow disappearance of the solvent from the polymer solution. In this case, the porosity is given by the solvent molecules in the film structure that diffuse outside of the film during the evaporation process [2,7]. At the same time, the polymer chains are entrained with the solvent from the base of the solution film to its surface, generating pores [34,35,36]. In the case of HA composite membranes, hydroxyapatite crystals appear both on the active and porous surfaces. These are observed both in a dispersed form and in the form of large crystals (agglomerates). As the amount of the HA particles in the film’s structure increases, large crystals have a higher volume [29,37,38]. Increasing the size of the crystals is a consequence of the poor dispersion of the inorganic filler in the polymer solution, the ultrasonication time being the same for all the samples. The presence of the HA on the porous surface can be explained by the weight of the particles, which are gravitationally deposited to the base of the polymer solution film. This behavior is also observed in the AFM.

**Figure 6:**
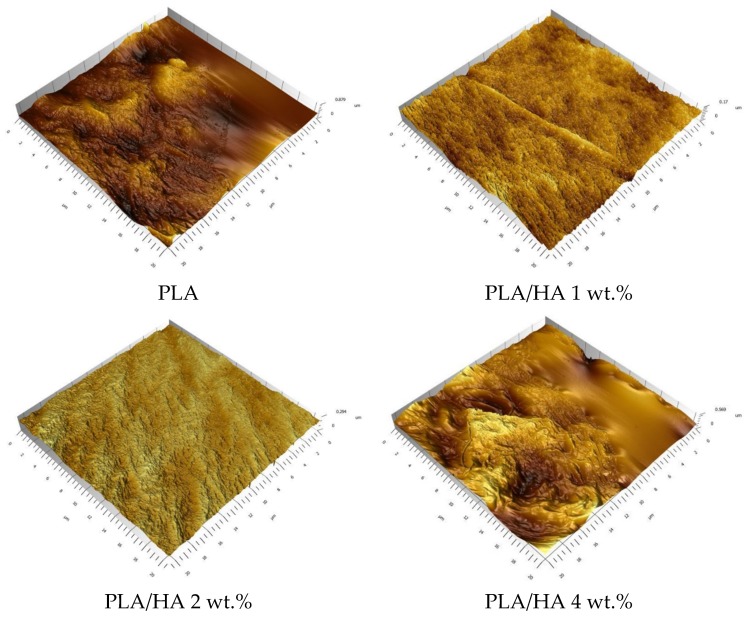
Atomic force microscopy (AFM) images of PLA and PLA/HA composite films.

The samples were morphologically characterized by atomic force microscopy in order to reveal the surface structure modification appearing with the variation of the composition. Figure 6 reveals the 3D morphology of the PLA and PLA/HA 4 wt.% samples. The PLA sample shows a surface topography with very high roughness areas and smooth areas. The morphological differences can be attributed to the polymer’s spatial arrangement in crystalline and amorphous zones. An ordered crystalline arrangement led to high roughness areas with an average roughness of about 41.1 nm, and a disordered amorphous arrangement led to a smooth area. The PLA/HA 4 wt.% composite film shows a decrease in the average roughness, with the average roughness being about 28.6 nm. This decrease appears because of the formation of HA aggregates on the membrane active surface, showing similar results to those observed in the SEM images. The above surface of the composite membrane reveals a more ordered structure due to a better HA dispersion with the average roughness decreasing to 10.8 nm.

The composite samples with 1 and 2 wt.% (Figure 6) highlight lower average roughness values of about 7.44 nm and 9.46 nm, respectively. The roughness decreasing assumes a more ordered surface structure due to a high HA dispersion. The higher HA dispersion for the composite samples with 1 and 2 wt.% HA loaded on can lead to the conclusion that these concentrations represent the right amount of inorganic phase within the polymeric matrix. Increasing the HA amount can overcome the system balance and generate HA aggregates.

**Figure 7:**
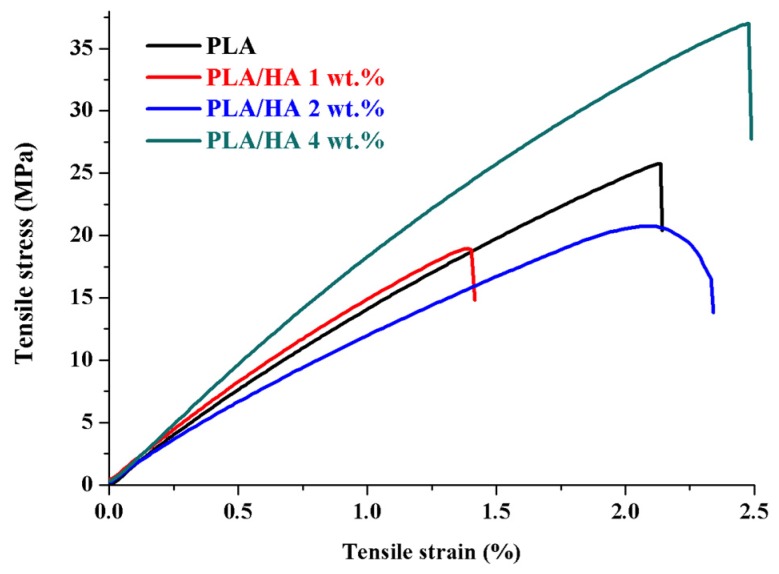
Mechanical tests of PLA and PLA/HA composite films.

Figure 7 displays tensile stress versus tensile strain curves for PLA and PLA/HA composite films. According to the figure and Table 2, a slight decrease in Young’s modulus in the case of the composite films with 2 and 4 wt.% HA loaded within the polymer matrices can be observed. This behavior may be explained on the one hand by the presence of HA, which acts as a local strain concentrator within the final material, and, on the other hand by the formation of the aggregates at 4 wt.% HA. The agglomeration of HA nanoparticles at 4 wt.% was also observed in SEM and AFM images and led to a low interfacial interaction between HA and the polymer. A similar trend has been observed by H.Y. Mi et al. In their study, Young’s modulus tended to decrease in the case of thermoplastic polyurethane/hydroxyapatite electrospun scaffolds with a concentration of >2 wt.% HA, and this was able to be explained by the nonuniform dispersion of HA within the polymer matrix, which led to an inhomogeneous stress distribution [39].

Previous reported research has shown an increased thermal resistance in the case of metal-doped HA with different cations like cerium [40], iron [41], zinc [42], and silver [43]. The higher thermal resistance is given in this case by the presence of cations inside the structure of HA, and also by the increased percentage of HA content (in all cases >10% wt.). Our observed small differences in the thermal behavior of the obtained films can be explained by the lower percent of HA (1–4% wt.), which is more suitable for potential application in osseointegration and is enough to influence the proliferation of pre-osteoblasts through the pores of polymeric films [5,7]. With potential application in osseointegration as films at the interface between a metallic implant and bone, porous films are preferred due to their more bioresorbable behavior under physiological conditions, with in this case the mechanical and thermal properties not being very important [8,9,10]. Higher mechanical properties can be obtained in the case of scaffolds obtained by 3D printing due to the large amount of polymer in the volume of the obtained material [44]. Also, in terms of mechanical properties, these can be significantly improved by the use of an additional polymer during the preparation of composite films, but with a much higher percent of HA. In the case of the composite film chitosan-PLA-HA at a 50% content HA amount at a compressive strength of 25,682 MPa, the strain to failure has been observed to be 70% with an elastic modulus of 857 MPa. With an increase in HA content (to 80%), the elastic modulus decreased to 660 MPa [45]. In these composites, PLA plays an important role, greatly influencing the nucleation and the growth of HA crystalline. An important factor in defining the mechanical and thermal properties is the synthesis method. When using cryomilling [46], high mechanical resistance and also improved thermal properties can be achieved via a Young’s modulus of the composite of 6 GPa and a compressive strength of 110 MPa, which are quite similar to the values for natural bone. In the case of electrospun fibers, the elastic modulus increase has been found to be approximately 40% for a system filled with micrometric HA (μHA) at 10%, 70% for systems of randomly oriented (R) PLA/μHA of 20%, 100% for a system with a nanometric randomly oriented HA (R PLA/nHA) of 10%, and up to 140% for an R PLA/nHA 20% composite [47]. All these methods assure a higher quantity of polymer respective to HA in the structure of composites, which can explain the higher mechanical properties in comparison with our films. Furthermore, even by solvent evaporation, asymmetric polymeric films are obtained with membrane structures which are characterized by a large free volume filled with air inside the material [1]. Osseointegration evaluation is more dependent on the synthesis method than other properties [48], being more suitable in the case of plasma discharge with sputter deposition at the surface of implants [49], Janus membranes [50], or membranes obtained by phase inversion/precipitation from PLA dissolved in acetone [51]. In comparison with membranes obtained from acetone by phase inversion, our films present the advantage of a very low diameter of pores, which is more suitable for further biomedical applications [25]. Also, the use of chloroform as a solvent can assure a better manipulation of pore diameter through the temperature of evaporation (a higher temperature will assure an increased speed of evaporation with a lower diameter of pores and a lower temperature of evaporation will decrease the speed of film formation and will also imply a lower pore diameter).

## 4. Conclusions

In this work, PLA/HA composite films were synthesized starting from a polymer solution in chloroform (12 wt.%) in which various concentrations of hydroxyapatite (1, 2, and 4 wt.% reportable to the polymer) were dispersed by ultrasonic methods, followed by the synthesis of membrane materials by solvent evaporation, resulting in polymeric composite films. The synthesized membranes were morphologically (by SEM and AFM) and structurally (by FT-IR and Raman spectroscopy) characterized, and the thermal behavior of the synthesized composite films was studied. SEM and AFM images showed the presence of micro-structured hydroxyapatite particles in the composite film structure, as well as changes in the surface morphology. There was a significant decrease in the crystallinity of the composite films compared to the pure polymer, this being explained by the decrease in the arrangement of the polymer chains and a concurrent increase in their degree of disorder. Also, the composite films were characterized in terms of their thermal behavior and it was observed that the presence of hydroxyapatite crystals did not have a significant influence on the degradation temperature of the composite films. Future research will study the influence of HA particle dimensions on composite polymer films using the same conditions (solvent and evaporation method) and in vitro tests related to the synthesized materials.

## Figures and Tables

**Table 1:** Thermal characteristics of obtained materials (where Tm1 and Tm2 are melting temperatures for low respectively high-temperature endotherms, ΔHc is crystallization enthalpy of the sample and ΔHm is melting enthalpy of the sample).

Sample Name	wt.%	Td10% (°C)	Tmax (°C)	Tc	Tm1	Tm2	ΔH_c_ (J/g)	ΔH_m_ (J/g)	χ_c_ (%)
PLA	100	221	352	106.9	141.4	148.4	18.99	21.82	3.02
PLA/HA 1 wt.%	99	226	350	103.9	142.6	150.3	22.63	23.3	0.72
PLA/HA 2 wt.%	98	274	351	104.2	142.6	150.6	22.63	23.78	1.23
PLA/HA 4 wt.%	96	254	351	106	141.8	149.5	24.75	26.37	1.73

**Table 2:** Mechanical tests of obtained materials.

Sample Name	Young’s Modulus (MPa)
PLA	17 ± 0.59
PLA/HA 1 wt.%	17 ± 0.55
PLA/HA 2 wt.%	15 ± 0.14
PLA/HA 4 wt.%	14 ± 1.44
